# The effects of pregnancy happiness on prenatal attachment and maternity function: a longitudinal study

**DOI:** 10.1590/1806-9282.20250040

**Published:** 2025-09-19

**Authors:** Sümeyye Barut, Nuray Kurt, Esra Sabanci Baransel

**Affiliations:** 1 Fırat University, Department of Midwifery – Elâzığ, Turkey.; 2 İnönü University, Department of Midwifery – Malatya, Turkey.

**Keywords:** Pregnancy, Happiness, Motherhood, Maternal-fetal relations

## Abstract

**OBJECTIVE:**

The aim of the study was to investigate the relationship between pregnancy happiness and prenatal attachment, as well as postpartum maternal functioning.

**METHODS:**

The study was conducted between December 2022 and January 2024 and involved 312 women. Data were collected using the Oxford Happiness Scale Short Form, Prenatal Attachment Inventory, and Barkin Maternity Function Scale. The Oxford Happiness Scale Short Form was administered during the second trimester of pregnancy, the Prenatal Attachment Inventory during the third trimester, and the Barkin Maternity Function Scale at 6–10 weeks postpartum.

**RESULTS:**

The study found that prenatal attachment mediates the effect of pregnancy happiness on maternal functioning. Additionally, it was observed that both pregnancy happiness and prenatal attachment have a direct impact on maternal functioning.

**CONCLUSION:**

This study clarifies the mechanism linking pregnancy happiness and maternal functioning. The findings suggest that increasing levels of pregnancy happiness and prenatal attachment may improve positive attitudes and ensure high maternal functioning.

## INTRODUCTION

Happiness is a concept that refers to an individual's cognitive evaluations of their life, including satisfaction, satisfaction with life, subjective well-being, and emotionally pleasant feelings^1^. Pregnancy is a happy event for both the woman and her family, but it is also a situation that increases emotional fluctuations in women^2^. The pregnancy period is characterized by unique physiological, psychological, and social changes that occur in each trimester. During the first trimester of pregnancy, the process of adapting to the reality of pregnancy takes place^3^. During the second trimester of pregnancy, the mother and baby begin to bond as the mother adapts to the pregnancy and the mother feels the baby's movements. The presence of the baby can bring the mother happiness. During the third trimester of pregnancy, it is common for women to experience negative emotions such as fatigue and fear of childbirth. These changes can have an impact on prenatal attachment, which is influenced by various factors^2,4^. Research has shown that negative emotional states, including depression, anxiety, stress, and unhappiness, may have a detrimental effect on prenatal attachment^3,5-7^.

Prenatal attachment also affects the relationship between mother and child throughout life, with the continuation of the positive conditions between mother and baby that begin with pregnancy^8^. The relationship between maternal competence and maternal function suggests that a mother's sense of competence toward her baby has a positive impact on the development of healthy attachment^9^. Research has shown that mother–infant attachment during the prenatal period has a significant impact on the mother. It motivates healthy behaviors, increases adaptation to the maternal role, and fosters a sense of responsibility for the baby's safety, nutrition, and psychological well-being. Additionally, it has a protective role against perinatal depression^10-12^.

Barkin defined postpartum functional status as the mother's ability to care for her baby, take care of herself, form an attachment with her infant, manage her emotions, adapt to her maternal role, and receive social support^13^. Once the baby is born and the mother begins to interact with them, she starts to recognize her maternal functions, duties, and responsibilities^14^. A positive bond can develop between a happy mother and her baby, who is ready to be a mother, loves her baby, and is expected to be more sensitive to her child's needs. The general purpose of this study is to examine the relationships between pregnancy happiness, prenatal attachment, and maternal function. In line with this general purpose, answers were sought to the following research hypotheses:

Hypothesis 1

Pregnancy happiness and prenatal attachment have a direct impact on maternal functions.

Hypothesis 2

Prenatal attachment has a mediating role between pregnancy happiness and maternal function.

## METHODS

**Figure 1:**
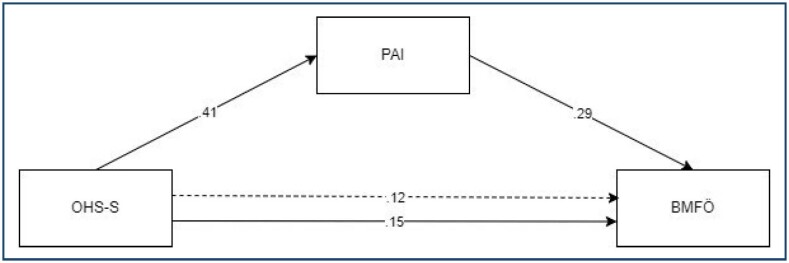
Standardized regression coefficients and path model.

The study utilized an analytical approach and developed a model based on longitudinal data, as shown in Figure 1. The model demonstrates a direct effect of pregnancy happiness and prenatal attachment on maternal function, with prenatal attachment serving as a mediating variable between pregnancy happiness and maternal function.

### Participants

This longitudinal study was conducted from December 2022 to January 2024. The sample size was calculated using OpenEpi version 3, a publicly available statistical software. The sample size was determined to be at least 385 with a 5% error level, a 95%CI at a two-sided significance level, and 80% power. However, incompletely filled, incomplete, or incorrectly coded surveys were identified, and the survey forms of 58 participants were excluded from the evaluation because they were considered invalid. Additionally, 15 people wanted to leave the study because they did not want to complete it. Thus, the study was completed with 312 people. The research was conducted with pregnant women who applied to the pregnancy training class at a university hospital. The study's inclusion criteria required participants to be 18 years of age or older and in their second trimester of pregnancy (14–26 weeks). Written informed consent was obtained from women who volunteered to participate in the study. Ethical approval for the research was obtained from the University's Health Sciences Non-Interventional Clinical Research Ethics Committee (Decision Number: 2022/11-21).

### Data collection

The researchers collected data from pregnant women who applied to the pregnancy education class in the hospital using face-to-face interviews. Survey forms were administered after obtaining participants’ consent. Data collection occurred in three stages. During Phase 1, we administered the "Personal Information Form and Oxford Happiness Scale Short Form" to pregnant women in their second trimester (between 14 and 27 weeks) who met the study's inclusion criteria and obtained their personal contact information. In the second stage, we contacted them by phone during their third trimester (between 28 and 41 weeks) and administered the "Prenatal Attachment Inventory." In the third stage, we contacted them 6–10 days after giving birth and administered the "Barkin Maternal Function Scale."

### Measures

Oxford Happiness Scale Short Form (OHS-S): The scale was developed by Hills and Argyle to measure happiness^15^. A high score obtained from the 29-item, 6-point Likert-type scale indicates that the level of happiness has increased. The Cronbach alpha value of the scale, whose Turkish validity and reliability study was conducted by Doğan and Çötok, was found to be 0.71^16^. In this study, the Cronbach's alpha reliability coefficient of the scale was found to be 0.79.

Prenatal Attachment Inventory (PAI): The scale was developed by Mary Muller in 1993 and adapted to Turkish by Yılmaz and Beji in 2009^17,18^. It is seen that the scale is used in its original form and in its Turkish version after the 20th week of pregnancy. Each item is a 4-point Likert type with a score between 1 and 4. A minimum score of 21 points is obtained from the scale, and a maximum score of 84 points is obtained. A high score indicates a high level of prenatal attachment. In the original study of the scale, Cronbach's alpha coefficient was found to be 0.84. In this study, the Cronbach's alpha reliability coefficient of the scale was found to be 0.83.

Barkin Maternity Function Scale (BMFS): The Barkin Maternity Function Scale was developed by Barkin in 2009 to measure the functional status of mothers after giving birth. According to Barkin, the Cronbach's alpha coefficient of the scale is 0.87^13^. Aydın and Kabukcuoğlu conducted a validity and reliability study of the Turkish version of the scale^19^. The scale's scoring ranges from 0 to 96, with lower scores indicating poorer functional status. In their study, the Cronbach's alpha reliability coefficient of the scale was calculated as 0.81.

### Data analysis

In the analysis of the data, SPSS 25 and AMOS 25 (Analysis of Moment Structures 25) package program used path analysis, a type of structural equation modeling, to examine the relationships between variables^20^. Before starting the path analysis, some preliminary analysis attempts and examinations were made. Preliminary analysis included correlations and descriptive statistics. In the model, the maternal function was included as the dependent variable, pregnancy happiness was included as the independent variable, and prenatal attachment was included as the mediating variable. Thus, we analyzed the data of 312 participants. The fit of parameters between the variables in the measurement model is chi-square/degrees of freedom (χ^2^/df), comparative fit index (CFI), and (TLI), and root-mean-square error of approximation (RMSEA). It was evaluated by examining the standardized root mean square residual (SRMR). To evaluate the fit values, the reference values specified in the literature were taken as criteria^21,22^. Additionally, analyses were carried out with the bootstrapping method with a sample size of 5,000. The bootstrap method is simple and reliable in non-parametric estimation methods^23^. In this context, analyses were carried out with the bootstrapping method with a sample size of 2,000 and a 95%CI. The absence of "0" among the confidence intervals shows that the direct and indirect effects are significant^24^.

## RESULTS

The mean age of the pregnant women was 28.39±5.23 years, with 70.8% of them being 26 or older. It was determined that 47.8% had a high school education or less, 78.8% were primiparous, and 85.9% wanted to be pregnant.

Table 1 presents the results of the correlation analysis and descriptive statistics, including mean, standard deviation, and minimum–maximum values. The analysis shows a moderate positive relationship (r=0.432) between OHS-S and PAI and a low positive relationship (r=0.273*) between OHS-S and BMFS.

**Table 1:** The relationship between Oxford Happiness Scale Short Form, Prenatal Attachment Inventory, and Barkin Maternity Function Scale.

	OHS-S	PAI	BMFS
OHS-S	–	r=0.432*	r=0.273*
Mean±SD	25.55±5.08	65.97±10.32	69.92±14.28
Min–max	10–70	28–84	5–90

* Correlation is significant at the 0.01 level (two-tailed).

OHS-S: Oxford Happiness Scale Short Form; PAI: Prenatal Attachment Inventory; BMFS: Barkin Maternity Function Scale; SD: standard deviation.

### Model testing

As a result of testing the proposed model with path analysis, it is seen that the goodness-of-fit values (χ^2^/df=2.26; RMSEA=0.054; SRMR=0.036; CFI=0.90; and TLI=0.95) obtained are good and acceptable.

Figure 1 presents the standardized regression coefficients and path model, while Table 2 shows the results of 5,000 sample bootstrapping analyses used to determine direct and indirect relationships between variables. The direct effects indicate that pregnancy happiness has a significant positive effect on prenatal attachment (β=0.415) and maternal function (β=0.149). Additionally, there is a positive and significant effect of prenatal attachment on maternal function (β=0.285). Furthermore, it seems that pregnancy happiness explains about 17% of the variance in prenatal attachment.

**Table 2:** Standardized direct, indirect, and total effects for variables explaining the Barkin Maternity Function Scale.

		95% BCI						
		B	SE	Z	Lower	Upper	p	R^2^
Direct effect								
	OHS-S→PAI	0.415	0.070	6.653	0.281	0.552	0.00	0.17
	OHS-S →BMFÖ	0.149	0.085	2.126	0.008	0.341	0.03	
	PAI→BMFÖ	0.285	0.076	4.080	0.139	0.434	0.00	
Indirect effect								
	OHS-S →PAI→BMFÖ	0.118	0.039	3.025	0.050	0.206	0.00	
	Total effect	0.267	0.090	2.966	0.100	0.451	0.00	0.14

OHS-S: Oxford Happiness Scale Short Form, PAI: Prenatal Attachment Inventory, BMFÖ: Barkin Maternity Function Scale, BCI: Bayesian credible interval; SE: standard error.

The study found that pregnancy happiness has a positive and significant effect on maternal function (β=0.118) through prenatal attachment, indicating that prenatal attachment acts as a mediator between pregnancy happiness and maternal function. The model explains approximately 14% of the variance in maternal function (Table 2).

## DISCUSSION

This study aimed to investigate the relationship between pregnancy happiness, prenatal attachment, and maternal function. Hypotheses were developed for the variables, and a model was proposed based on theoretical explanations and obtained results. Path analysis results indicated that the model had good fit values, and all hypotheses were confirmed. The findings contribute to understanding the factors that affect pregnancy happiness and prenatal attachment in explaining maternal function.

The primary objective of our research is to investigate the correlation between pregnancy happiness and other variables, such as prenatal attachment and maternal function. The study revealed that pregnancy happiness and prenatal attachment have a significant positive impact on maternal function. This implies that higher levels of pregnancy happiness and prenatal attachment are associated with better maternal functioning. Pregnancy happiness was found to have a significant direct effect on prenatal attachment (β=0.415) and maternal function (β=0.149). Additionally, prenatal attachment significantly predicted maternal function (β=0.285), confirming that prenatal attachment acts as a mediator between pregnancy happiness and maternal function. The model explained about 14% of the variance in maternal function, with pregnancy happiness explaining approximately 17% of the variance in prenatal attachment. This indicates that while pregnancy happiness is a strong predictor, other unexamined variables may also contribute to maternal function, suggesting potential areas for further research. No studies have been found in the literature that examine the relationship between pregnancy happiness, prenatal attachment, and maternal function characteristics. Most of the conducted studies predict maternal function in the postpartum period^13^. In their study, Gholizadeh Shamasbi et al. found a relationship between mental health and maternal function at 1 month postpartum^25^. Aydın's study, like ours, supports the idea that the pregnancy process affects maternal function^19^. Other studies have shown that negative emotional states during pregnancy, such as unhappiness, may lead to difficulties bonding with the child both during and after birth, ultimately reducing overall well-being^26-28^. This information reinforces the findings of our study. Pregnant women who maintain a positive psychological state tend to focus on their unborn babies and experience the pregnancy process happily. Therefore, a positive approach during pregnancy can lead to higher attachment scores. To increase prenatal attachment scores, it is important to focus on the baby and have positive thoughts. Completing a healthy pregnancy is believed to significantly reduce postpartum psychological distress and allow for a focus on motherhood.

Another finding of our study is related to the second hypothesis of our research. Our study revealed that pregnancy happiness indirectly impacts maternal function through prenatal attachment, supporting the development of a positive attitude toward the fetus and increasing adaptation to postpartum maternal function. The indirect effect of pregnancy happiness on maternal function through prenatal attachment (β=0.118) supports the idea that emotional well-being during pregnancy can foster better adaptation to postpartum maternal functions. Oruç and Kukulu's study evaluated maternal function and mother–infant attachment in the postpartum period^9^. The study found a positive, low, and significant relationship between attachment and self-care, maternal psychology, baby care, social support, and maternal adjustment scores. Mothers adapt to their new roles by becoming aware of their babies and their physical, emotional, and social recovery in the postpartum period. During this period, the mother's interaction with her baby supports attachment. This information supports our hypothesis.

As it can be understood from these findings;

This study suggests that emotional well-being during pregnancy can lead to a stronger bond and interaction between pregnant women and their unborn babies, which can facilitate their adaptation to postpartum maternal functions. This information highlights the positive impact of high pregnancy happiness on maternal function and explains the indirect effect of pregnancy happiness on maternal function through prenatal attachment.

### Limitations

This study has some limitations. First, due to the cross-sectional nature of the research, it is not possible to infer causal relationships from the results. Second, the data collected were based on self-reports from women and on the internet, which may have led to over- or under-reporting of responses and could have biased our conclusions. Future studies may benefit from utilizing alternative data collection methods. Due to the regional expansion of the study in a specific region, the impact of cultural differences can be seen as a separate limitation. Additionally, as this study only included women, it is unclear whether the findings can be generalized to other populations.

## CONCLUSION

In summary, this study examined the pathway between pregnancy happiness and prenatal attachment and maternal function in women. As a result, we concluded that the effect of pregnancy happiness on maternal function is mediated by prenatal attachment and that pregnancy happiness and prenatal attachment directly affect maternal functions. Experimental and interventional studies on the subject may provide additional evidence on the subject.

## Data Availability

The datasets generated and/or analyzed during the current study are available from the corresponding author upon reasonable request.
